# Nitrogen Allocation Tradeoffs Within-Leaf between Photosynthesis and High-Temperature Adaptation among Different Varieties of Pecan (*Carya illinoinensis* [Wangenh.] K. Koch)

**DOI:** 10.3390/plants11212828

**Published:** 2022-10-24

**Authors:** Qiwen Xu, Huichuan He, Binghui He, Tianyang Li, Yumin Liu, Shunyao Zhu, Gaoning Zhang

**Affiliations:** College of Resources and Environment, Southwest University, Chongqing 400715, China

**Keywords:** nitrogen allocation tradeoffs, homeostatic balance, variety identification, physiological properties

## Abstract

Interpreting leaf nitrogen (N) allocation is essential to understanding leaf N cycling and the economy of plant adaptation to environmental fluctuations, yet the way these mechanisms shift in various varieties under high temperatures remains unclear. Here, eight varieties of pecan (*Carya illinoinensis* [Wangenh.] K. Koch), Mahan, YLC10, YLC12, YLC13, YLC29, YLC35, YLJ042, and YLJ5, were compared to investigate the effects of high temperatures on leaf N, photosynthesis, N allocation, osmolytes, and lipid peroxidation and their interrelations. Results showed that YLC35 had a higher maximum net photosynthetic rate (*P*_max_) and photosynthetic N-use efficiency (PNUE), while YLC29 had higher N content per area (*N*_a_) and lower PNUE. YLC35, with lower malondialdehyde (MDA), had the highest proportions of N allocation in rubisco (*P*_r_), bioenergetics (*P*_b_), and photosynthetic apparatus (*P*_p_), while YLC29, with the highest MDA, had the lowest *P*_r_, *P*_b_, and *P*_p_, implying more leaf N allocated to the photosynthetic apparatus for boosting PNUE or to non-photosynthetic apparatus for alleviating damage. Structural equation modeling (SEM) demonstrated that N allocation was affected negatively by leaf N and positively by photosynthesis, and their combination indirectly affected lipid peroxidation through the reverse regulation of N allocation. Our results indicate that different varieties of pecan employ different resource-utilization strategies and growth–defense tradeoffs for homeostatic balance under high temperatures.

## 1. Introduction

As sessile organisms, the growth and reproduction of plants are limited inherently by external environmental stresses, including biotic and abiotic stresses [1]. Among ever-changing components of the environment, the constantly rising ambient temperature is regarded as one of the most detrimental stresses [2], as it will trigger a series of abiotic stresses, such as drought stress and solar radiation, along with disease outbreaks [3,4]. Elevating air temperatures and summer drought events are increasing in frequency due to the changing climate. The global air temperature is predicted to rise by 0.2 °C per decade, which will lead to temperatures 1.8–4.0 °C higher than the current level by 2100 [5,6]. This is drawing attention from scientists in environmental, ecology, and genetics fields as the growth, metabolism, quality, and yield of plants are substantially affected, directly or indirectly, and often lethally, by the modification of surrounding environmental components [7,8]. Therefore, a comprehensive understanding of plant environmental adaptability is indispensable to mitigate the adverse effects of high temperatures on plants.

To survive stresses, plants have evolved sophisticated defense mechanisms against environmental stresses, which are fundamental for life development and resilience to even hostile environments [9]. Photosynthesis serves as a sensor of environmental stress achieved across different homeostatic mechanisms [10]. Meanwhile, photosynthesis is highly sensitive to high-temperature stress and is often affected before other leaf-cell functions are impaired [11]. For instance, high temperatures not only inhibit the function of PSII, impede electron-transport rates, decrease rubisco activase, and degrade chlorophyll content, but also possibly induce membrane permeability and directly damage the chloroplast thylakoid membranes, which further lowers light harvesting, photosynthetic capacity, and photosynthetic nitrogen-use efficiency (PNUE) [2]. Nevertheless, adaptable plants can show extensive competence to fine-tune their photosynthetic characteristics to their growth temperatures, which regulate the accumulation of osmolytes or cell redox homeostasis, such as with soluble protein, soluble sugar, free amino acids (particularly proline) and glutathione [1,2], demonstrating that there are tradeoffs between leaf photosynthesis and environmental adaptability. However, how these tradeoffs vary with different varieties, especially in the crucial stage of reproduction, is largely unclear.

Nutrient cycling is an important process for homeostasis in plant cells, which not only regulates the tight control of nutrient concentration, but may also allow the utilization of external nutrients or the dissipation of excess energy without a greater expense [9]. Nitrogen (N) is regarded as one of the most important nutrient elements both in agricultural and natural environments, determining leaf functional traits via nutrient cycling and allocation among photosynthetic machinery, functional proteins, and structural compounds [12,13,14]. Plants typically invest about 75% of leaf N in chloroplasts for photosynthesis, which is largely affected by environmental fluctuations [15,16]. N allocation between photosynthetic and non-photosynthetic proteins mirrors a tradeoff of productivity and environmental adaptability [17,18,19]. More leaf N investment in the fraction of structural proteins (e.g., cell-wall binding proteins) reduces photosynthetic capacity and PNUE while increasing the persistence of leaf life [18,20]. Besides this, leaf N in photosynthetic apparatus consists of N allocation to light-harvesting (N in light-harvesting chlorophyll–protein complexes), to bioenergetics (total N content of cytochrome f, ferredoxin NADP reductase, and the coupling factor), and to rubisco. Plants can mediate this large N pool inside leaves to enhance resistance and tolerance to environmental changes, such as drought stress [19], salt stress [13], irradiance changes [21], and elevated atmospheric CO_2_ concentrations [22,23]. So far, many studies have performed tests on the effects of stress conditions on tradeoffs in within-leaf strategies without high-temperature stress, and thus high-temperature stress effects need to be urgently studied.

Pecan (*Carya illinoinensis* [Wangenh.] K. Koch) is an economically important nut tree of the Carya genus, which is native to the United States and Mexico [24]. It is widely grown for its highly nutritious kernels and resilient wood in 20 countries on five continents, such as China, Iran, Turkey, and Australia, over a total area of between 96,909 and 390,924 ha, with a clear upward trend [25]. Throughout those habitats, pecans generally develop under a range of climatic conditions with complicated and volatile temperature ranges and cumulative precipitation during the growing season [24,26]. Meanwhile, climate change has also been altering the situation and a growing number of reports have been published on various responses of pecan to environmental factors to understand its decreased growth or survival [24,27,28]. Variety identification, including dramatic phenotypic and physiological diversifications, is one of the crop-domestication processes for increasing yield and high-quality crops [29]. Advantaged crops may have efficient adaptive strategies and nutrient cycling, that is, higher leaf N content and net photosynthesis combined with strong tolerances for stress in order to yield under conditions of high resource availability [20,30]. Hence, we selected eight varieties of pecan to examine resource-utilization and adaptive strategies under high temperatures. We aimed to: (1) compare differences in leaf N, photosynthesis, N allocation, osmolytes, and lipid peroxidation from eight varieties and (2) establish a network relationship among those indicators in pecans under high temperatures.

## 2. Results

### 2.1. Daily Temperature Records

**Figure 1:**
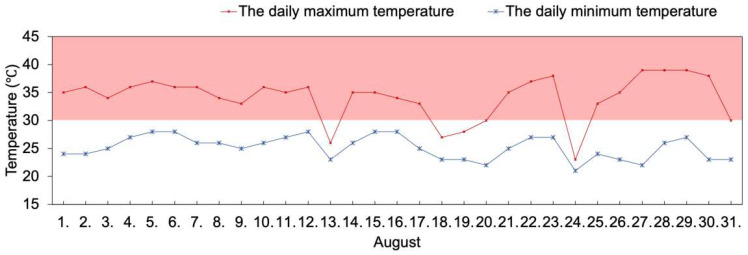
Daily minimum temperature and daily maximum temperature at the study site in August.

The daily minimum and maximum temperature were recorded in August (Figure 1). The daily minimum temperature was 21–28 °C. There were 27 days in August on which the daily maximum temperature was above 30 °C, i.e., all except the 13th, 18th, 19th, and 24th, and there was 19 days that the daily maximum temperature was above 35 °C.

### 2.2. Differences in Leaf Nitrogen and Photosynthesis from Eight Varieties under High Temperatures

**Figure 2:**
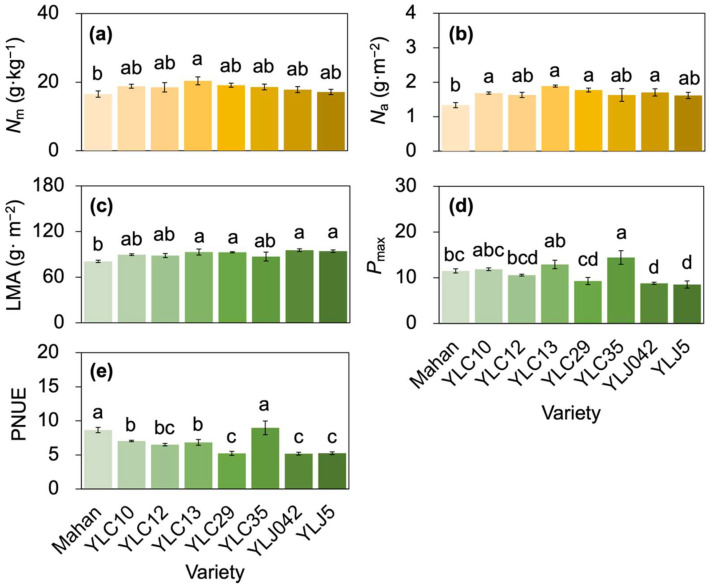
Histograms showing the differences in *N*_m_ (**a**), *N*_a_ (**b**), LMA (**c**), *P*_m_ (**d**), and PNUE (**e**) from eight varieties. Different lowercase letters above the bars indicate significant differences among eight varieties with *p* < 0.05. *N*_m_, nitrogen content per mass; *N*_a_, nitrogen content per area; LMA, leaf mass per area; *P*_max_, maximum net photosynthetic rate; PNUE, photosynthetic nitrogen-use efficiency.

Among the eight varieties, leaf N content per mass (*N*_m_) and N content per mass (*N*_a_) were the highest in YLC13, 23.34% (*N*_m_) and 41.46% (*N*_a_) higher than those in Mahan with the lowest values (Figure 2a,b). Simultaneously, *N*_a_ was 26.2%, 41.5%, and 32.8% higher in YLC10, YLC 29, and YLJ042 than those in Mahan (*p* < 0.05), respectively (Figure 2b). PNUE, on the contrary, showed a higher level in Mahan and YLC35 but a lower level in YLC29, YLJ042, and YLJ5, respectively (*p* < 0.05; Figure 2e). Leaf mass per area (LMA) was higher in YLC13, YLC29, YLJ042, and YLJ5, and was the lowest in Mahan (*p* < 0.05; Figure 2c). The maximum net photosynthetic rate (*P*_max_) was the highest in YLC35 (14.43 ± 1.49), and lower in YLJ042 (8.78 ± 0.24) and YLJ5 (8.52 ± 0.78) (*p* < 0.05), respectively (Figure 2d).

### 2.3. Changes in Leaf Nitrogen Allocation of Eight Varieties under High Temperatures

**Figure 3:**
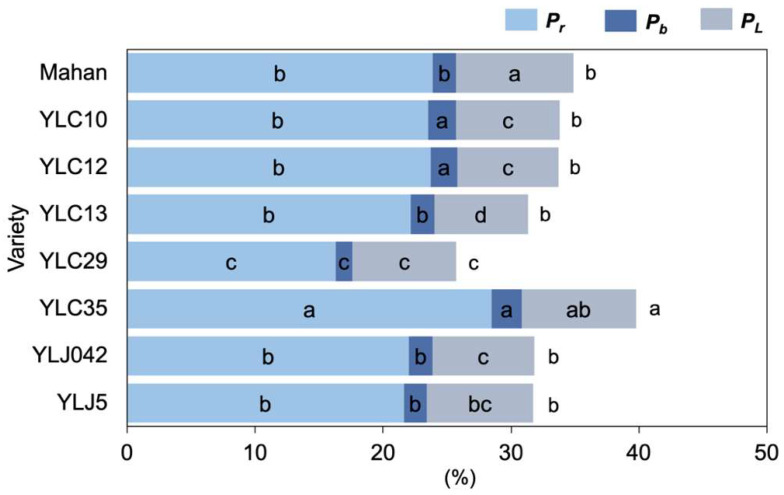
Stacked histogram showing differences in the proportion of leaf nitrogen allocation from eight varieties. Different lowercase letters above the bars indicate significant differences among eight varieties with *p* < 0.05. *P*_r_, the proportions of leaf nitrogen allocation in rubisco; *P*_b_, the proportions of leaf N allocation in bioenergetics; *P*_L_, the proportions of leaf nitrogen allocation in light-harvesting; *P*_p_, the proportions of leaf nitrogen allocation in photosynthetic apparatus, was recorded by the total length of bars.

The proportion of N allocation within leaves varied among the eight varieties (Figure 3). There was a similar trend between the proportions of leaf nitrogen allocation to rubisco (*P*_r_) and bioenergetics (*P*_b_): the highest proportion was found in YLC35 while the lowest proportion was recorded in YLC29 (*p* < 0.05), increasing by 75.05% and 74.73%, respectively. In addition, the *P*_b_ in YLC10 and YLC12 was not significantly different from YLC35, and was significantly higher than that in other varieties (*p* < 0.05). Meanwhile, Mahan had the highest proportions of leaf N allocation to light-harvesting (*P*_L_), followed by YLC35, and the lowest YLC13 (*p* < 0.05). Taken together, the proportion of nitrogen allocation to photosynthetic apparatus (*P*_p_) was the highest in YLC35, followed by Mahan, YLC10, YLC12, YLJ042, YLJ5, and YLC12, and was lowest in YLC29 (*p* < 0.05).

### 2.4. Responses of Osmolytes’ Accumulation and Lipid Peroxidation to High Temperatures

**Figure 4:**
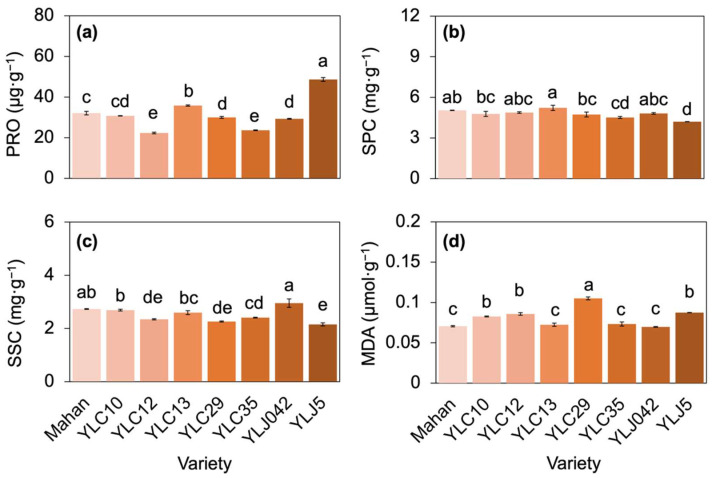
Histogram showing differences in the content of osmolytes [PRO (**a**), SPC (**b**), SSC (**c**)] and lipid peroxidation [MDA (**d**)] from eight varieties. Different lowercase letters above the bars indicate significant differences among eight varieties with *p* < 0.05. PRO, proline; SPC, soluble protein content; SSC, soluble sugar content; MDA, malondialdehyde.

Proline (PRO) in the eight varieties was in the order YLJ5 > YLC13 > Mahan > YLC10 > YLC29 and YLJ042 > YLC12 and YLC35, therein, the value in YLJ5 was 117.87% and 105.56% higher than the values in YLC12 and YLC35, respectively (*p* < 0.05; Figure 4a). Conversely, soluble protein content (SPC) and soluble sugar content (SSC) were the lowest in YLJ5 (Figure 4b,c). The highest SPC and SCC were found in YL13 and YLJ042, respectively, which were 24.25% and 37.16% higher than those in YLJ5, respectively (*p* < 0.05; Figure 4b,c). Malondialdehyde (MDA) was the highest in YLC29 (0.11 ± 0.002 μmol·g^−1^), which was 20.16%, 22.52%, and 27.11% higher than the medium values in YLJ5, YLC12 and YLC10, and 43.41%, 45.09%, 48.85%, and 50.69% higher than the lower values in YLC35, YLC13, Mahan, and YLJ042, respectively (*p* < 0.05; Figure 4d).

### 2.5. Principal Component Analysis

**Figure 5:**
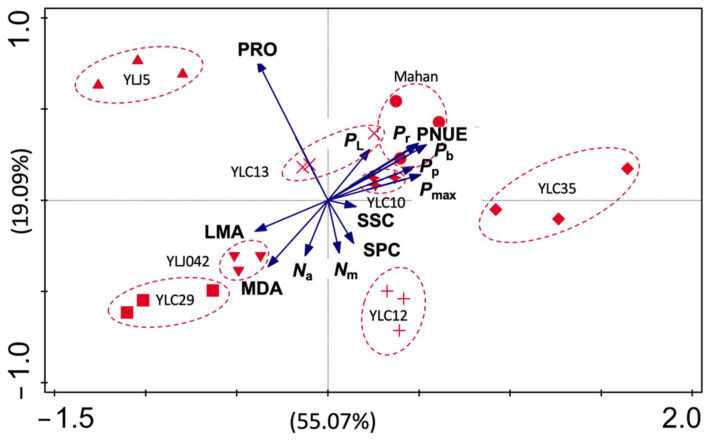
A principal component analysis (PCA). *N*_m_, nitrogen content per mass; *N*_a_, nitrogen content per area; *P*_max_, maximum net photosynthetic rate; PNUE, photosynthetic nitrogen-use efficiency; *P*_r_, the proportions of leaf nitrogen allocation in rubisco; *P*_b_, the proportions of leaf N allocation in bioenergetics; *P*_L_, the proportions of leaf nitrogen allocation in light-harvesting; *P*_p_, the proportions of leaf nitrogen allocation in photosynthetic apparatus; PRO, proline; SPC, soluble protein content; SSC, soluble sugar content; MDA, malondialdehyde.

Principal component analysis (PCA) showed that axis 1 mostly explains 55.07% of the total variance and axis 2 explains 19.09%; thus, taken together, they explain 74.16% of the total (Figure 5). PCA additionally revealed the clear separation between varieties of pecan based on the measured indicators. Eight varieties were divided into four groups: Mahan, YLC13, and YLC10 in the first quadrant; YLJ5 in the second quadrant; YLC29 and YLJ042 in the third quadrant; YLC12 in the fourth quadrant (Figure 5).

### 2.6. Interrelations

**Figure 6:**
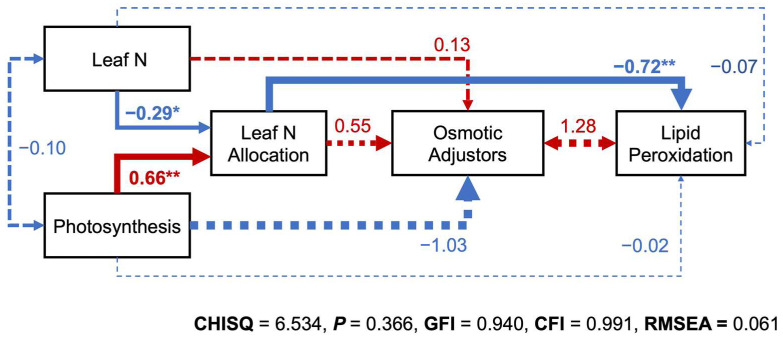
Structural equation models (SEM) showing potential causal effects of leaf nitrogen and photosynthesis on nitrogen allocation, osmotic adjustors, and lipid peroxidation. Arrow thickness was scaled proportionally to the standardized path coefficients (numbers on arrows). Continuous red and continuous blue arrows indicate positive and negative relationships, respectively, whereas dashed arrows indicate no significant relationship. *, *p* < 0.05; **, *p* < 0.01.

Structural equation modeling (SEM) showed that leaf N had a direct negative effect on N allocation with 0.29 path coefficient (*p* < 0.05; Figure 6). Photosynthesis, on the other hand, accelerated leaf N allocation directly (*p* < 0.01) with a 0.66 path coefficient (Figure 6). PCA demonstrated that *P*_L_, *P*_r_, *P*_b_, and *P*_p_ had positive relationships with PNUE and *P*_max_, and negative relationships with *N*_m_ and *N*_a_, respectively (Figure 5). Moreover, leaf N and photosynthesis could indirectly affect lipid peroxidation through reverse regulatory processes of leaf N allocation, with a 0.72 path coefficient (*p* < 0.01; Figure 6). MDA under PCA also had positive relationships with *N*_m_ and *N*_a_ and negative relationships with *P*_max_ and PNUE (Figure 5).

## 3. Discussion

### 3.1. Resource Conservative and Acquisitive Strategies

In this study, August was a sensitive time of reproductive development in southwest China, in which the eight varieties were exposed to high temperatures (Figure 1), and a few degrees elevation in temperature during the fruiting time could lead to the loss of entire plant cycles [2]. Under various adverse environmental stresses and high resource availability, plant strategies could have gradually shifted from more resource-conservative to more resource-acquisitive [31]. Here, although YLC29, YLJ042, and YLJ5 had higher *N*_a_, their PNUE under stress was the lowest. Mahan had the lowest *N*_a_ and medium *P*_max_ and YLC35 had higher *N*_m_ and the highest *P*_max_, yet they all presented the highest PNUE (Figure 2), echoing that adaptable varieties tend to have lower LMA, higher *P*_max_, and PNUE in order to grow faster and yield more [32,33]. Kebert et al. [4] also suggested that plant thermotolerance could be revealed by the maintenance of photosynthetic functionality. This may demonstrate that Mahan and YLC35 triggered a shift of plant traits along the leaf economic spectrum towards faster growth strategies involving higher photosynthetic capacity and N utilization, while YLC29, YLJ042, and YLJ5 had lower tolerance even though more N was absorbed. The changes in leaf N allocation further supported this hypothesis, in that YLC35 had the highest *P*_r_, *P*_b_, and *P*_p_, and higher *P*_L_. Mahan had the highest *P*_L_ and higher *P*_p_, compared with other varieties (Figure 3). The simultaneous increases in *P*_r_, *P*_b_, and *P*_L_ imply that for an efficient photosynthetic system there are coordinated associations between light-energy capture, light-energy transfer, and carbon assimilation [33].

### 3.2. Leaf N Allocation Reflected the Growth-Defense Tradeoffs

Growth–defense tradeoffs commonly occur in plants triggered by environmental restrictions, and their function in optimizing growth and defense depends jointly on internal and external factors [13]. In this study, pecans allocated 25.72–39.76% of leaf N to the photosynthetic apparatus (*P*_p_, Figure 3), which was lower than the result in C3 plants (≥50%) [14,34]. The difference may result from chlorophyll degradation at high temperatures [34] and accompanying higher solar radiation intensity [35], because leaf N invested in photosynthesis could be affected by species and environment, ranging from 30–70% [36]. For photosynthetic capacity, rubisco is the key enzyme in charge for carboxylation [16,37], contributing to *P*_r_ and accounting for a major portion of *P*_p_ in the eight varieties of pecan (Figure 3). It is an important strategy that absorbing more N in rubisco as well as increasing N investment in carboxylation can maintain a higher PNUE to withstand environmental stress [13,19,38]. Alternatively, numerous studies demonstrated that LMA could be considered as an indication of leaf structural tissues belonging to non-photosynthetic apparatus [18,37]. Our result showed YLC35 with lower LMA and the highest *P*_r_, *P*_b_, and *P*_p_, but YLC29 with the highest LMA and the lowest *P*_r_, *P*_b_, and *P*_p_ (Figure 2 and Figure 3), which implied that pecans regulated the tradeoff to ensure more leaf N to allocate to non-photosynthetic apparatus for enhancing tolerance, rather than to photosynthesis apparatus for boosting PNUE under high-temperature stress, reflecting adaptive strategies that display more activity in severely stressed plants [13,19].

### 3.3. Tolerance to High Temperatures May Rely on Compensation Mechanisms

The occurrence of oxidative stress in leaves under adversity explains the resistance and tolerance of the eight varieties. High temperatures can induce oxidative stress through the peroxidation of membrane lipids and cause disruption of cell-membrane stability by protein denaturation [2]. MDA accumulation is a marker of lipid peroxidation and membrane damage [39]; therefore, the eight varieties in this study were divided into three groups of injury levels: light injury in YLC35, YLC13, Mahan, and YLJ042; medium injury in YLJ5, YLC12, and YLC10; serious injury only in YLC29 (Figure 4d). Besides MDA, one of the key adaptive mechanisms is accumulations of amino acids, sugars, sucrose, and amines as osmotic adjustors to support plants to sustain growth in abiotic conditions [40]. Our result showed that YLJ5 had the highest PRO yet had the lowest SPC and SSC, and YLJ042 had lower PRO while having the highest SPC and SSC (Figure 4a–c). This implied that plant tolerance to high temperatures may rely on compensation mechanisms [4]; that is, the eight varieties selectively increased the levels of adjustors in the same protective systems for reducing membrane damage depending on their varietal characteristics. Therefore, the level of MDA, together with different osmotic parameters at high temperatures, suggested an adaptation to stress for the test varieties.

### 3.4. Tradeoffs between Growth and Defense Varied with Varietal Characteristic

In this study, SEM illustrated a positive effect of photosynthesis on leaf N allocation, echoing previous studies [13,19], but a negative effect of leaf N on N allocation in the photosynthetic apparatus in accordance with PCA, which was unexpected (Figure 6). N is an essential nutrient to facilitate plant growth and development [12]; however, Zhong et al. [19] also found that rice plants grown in high N conditions reduced N investment in photosynthesis under drought stress. A possible reason for the negative effect was that plants need to balance growth and tolerance under environmental fluctuations, namely transferring N to non-photosynthetic components to enhance their resistance at the cost of PNUE [18,19,41]. This corresponds with the indirect influence of leaf N and photosynthesis on lipid peroxidation through the adverse effect of leaf N allocation in our study, suggesting that N invested in non-photosynthetic proteins was a priority to enable more nutrients to repair leaf cell-membrane damage, even though this could lower plants’ growth and yield under high temperatures. This network relationship was generally established in these eight varieties, whereas the strategies were modulated by varietal characteristics, such as resistances and tolerances. This was supported by the PCA result that YLJ5, YL29, and YLJ042 were separated from other varieties on the same side of a quadrant (Figure 5). Based on the physiological responses to high temperatures, YLJ5, YL29, and YLJ042 tended to employ resource-conservative strategies and defense tradeoffs compared with other pecan varieties. However, differences of varietal characteristics require more comprehensive evidence, not only evaluating costs per unit leaf area, nutrient concentrations, and tissue turnover, but also establishing a sophisticated modeling approach to identify the complexity of plant homeostatic mechanisms under stress conditions. Meanwhile, molecular biology and biotechnology, such as transcriptome and metabolomic analysis, are also strong tools for in-depth research and exploration. We will further obtain molecular-level information and carry out a modeling approach allowing a quantitative assessment of the energy costs of high-temperature adaptation among various varieties to guide breeding and the engineering of molecular components.

## 4. Materials and Methods

### 4.1. Study Area and Plant Material

This study was conducted in Yongxin Town (106.50° E, 28.99° N), Qijiang District, Chongqing Municipality City, Southwest China. The elevation is 460–480 m above sea level and the aspect of the slope is southwest-facing. The study site has a typical subtropical monsoon climate with an average annual rainfall of 1 037.3 mm, and an annual average temperature of 17.5 °C. The overlying soil is mainly classified as yellow soil corresponding to mollic Inceptisols, according to the United States Department of Agriculture (USDA) Taxonomy. Eight varieties of pecan (*Carya illinoinensis* [Wangenh.] K. Koch), Mahan, YLC10, YLC12, YLC13, YLC29, YLC35, YLJ042, and YLJ5, were used as experimental materials with 5-year-old pecan seedlings (Table S1). These plants belonged to tree-cropping systems that were managed by local farmers and were randomly distributed on the same hillslope. Every plot with three replications was set up with five pecans of the eight varieties and the spacing and row spacing were 4 m. The leaves were measured and collected in August 2 022 as at that reproductive developmental stage plants are more sensitive to high temperatures than at the vegetative developmental stage [2].

### 4.2. Measurements of Leaf Gas Exchange

During a sunny day in mid-August, gas exchange was measured on fully expanded compound leaves of the 2nd or 3rd pair from below using a Li-6800 portable photosynthesis system (Li-Cor Inc., Lincoln, NE, USA) from 7:00 h to 19:00 h, in which period the photosynthetic state of plants is fully activated. The leaf-chamber parameters were as follows: 55% relative humidity of the air, 30 °C leaf temperature, 400 μmol·mol^−1^ CO_2_ concentration, 11 gradients of photosynthetically active radiation intensity (0, 20, 50, 80, 100, 200, 300, 600, 1000, 1500, 2000 μmol·m^−2^ s^−1^). The leaves were labeled, and leaf areas were calculated based on the labeled area. The following gas-exchange measurement was also conducted with the same labeled leaves. After these measurements, the net photosynthesis rate/CO_2_ (A-Ci) curves were measured with the above leaf-chamber parameters with a series of CO_2_ concentration gradients (400, 300, 200, 100, 50, 400, 600, 800, 1000, 1200, 1500 μmol·mol^−1^). All data were recorded when the leaves reached a photosynthetic steady state. The maximum net photosynthetic rate (*P*_max_), maximum electron transport (*J*_max_), and maximum carboxylation rate (*V*_cmax_) were calculated according to Sharkey et al. [42] based on the model developed by Farquhar et al. [43].

### 4.3. Determinations of Leaf Chlorophyll Content, Leaf Mass per Area, and Nitrogen Content

Leaf chlorophyll content was measured on fresh leaves which were extracted in 80% (*v*/*v*) acetone at room temperature in the dark until they turned white, and were then determined at 470, 645, and 663 nm for the absorbance of the extraction solution to calculate chlorophyll content per mass (Cc, mg·g^−1^). The leaf area (LA, m^2^) was scanned on fresh leaves by the Intelligent leaf-area measurement system (Top Cloud-agri Technology Co., Ltd., Hangzhou, China), and then the leaves were oven-dried at 80 °C for at least 48 h and their dry mass obtained (DM, g). The leaf mass per area (LMA) was calculated using the ratio of DM to LA. Leaf nitrogen content (*N*_m_, g·kg^−1^) was measured on leaves oven-dried by the Kjeldahl digestion procedure. Nitrogen content per area (*N*_a_) was calculated using the equation *N*_a_ = *N*_m_ × LMA.

### 4.4. Calculations of Leaf Photosynthetic Nitrogen-Use Efficiency and Nitrogen Allocation

PNUE was calculated as the ratio of *P*_max_ and *N*_a_. The leaf N allocation in the photosynthetic apparatus was divided into three categories: light-harvesting (N in light-harvesting chlorophyll–protein complex), bioenergetics (total N content of cytochrome f, ferredoxin NADP reductase, and the coupling factor), and rubisco. The proportions of leaf N allocation in the photosynthetic apparatus (*P*_p_), light-harvesting (*P*_L_), bioenergetics (*P*_b_), and rubisco (*P*_r_) were calculated as follows [44,45]:

$$P_{L}\;=\;\frac{C_{c}}{C_{B}\;\times \;N_{m}};$$

$$P_{b}\;=\;\frac{J_{max}}{8.06\;\times \;J_{\mathrm{mc}}\;\times \;N_{a}};$$

$$P_{r}\;=\;\frac{V_{cmax}}{6.25\;\times \;V_{\mathrm{cr}}\;\times \;N_{a}};$$

$$P_{p}\;=\;P_{L}\;+\;P_{b}\;+\;P_{r},$$

where *C*_B_ is the chlorophyll binding to PSI, PSII, and LHCII, determining how efficiently the nitrogen invested in thylakoids participates in light harvesting; *J*_mc_ is the maximum electron-transport rate; *V*_cr_ is the maximum rate of RuBP carboxylation per unit of rubisco protein; 6.25 [g rubisco (g nitrogen in rubisco)^−1^] is the conversion coefficient from nitrogen content to protein content, and 8.06 [μmol cyt f (g nitrogen in bioenergetics)^−1^] is the nitrogen content of rate-limiting protein and the molar stoichiometry relative to cyt f.

### 4.5. Assay of Osmolytes’ Accumulation and Lipid Peroxidation

Osmolytes included proline (PRO), soluble protein, and soluble sugar. PRO concentration was estimated using the acid-ninhydrin method [46]. Soluble protein content (SPC) was quantified through the Coomassie brilliant blue method [47]. Soluble sugar content (SSC) was determined by the sulfuric acid-anthrone colorimetry method [48]. Lipid peroxidation was determined through measurement of the malondialdehyde (MDA) using the thiobarbituric acid colorimetric method [49].

### 4.6. Statistical Analysis

A Shapiro-Wilk test was used to examine the normality of variables and the mean centering or log transformation was used as usual to ensure normality when the data are not normally distributed. Levene’s test was used to determine the homogeneity of variances. One-way analysis of variance (one-way ANOVA) was used to determine the differences in study variables between eight varieties, and comparison between means was determined using the Duncan’s multiple range test, *p* < 0.01 or *p* < 0.05 in IBM SPSS 25.0 software (SPSS Inc., Chicago, IL, USA). Principal component analysis (PCA) was forced to determine the relationship of various physiological indicators using Canoco 5.0 (Microcomputer Power, Ithaca, NY, United States), and Kaiser-Meyer-Olkin (KMO) and Bartlett’s tests were conducted to examine the suitability of the data for PCA. Structural equation modeling (SEM) was used to determine the direct and indirect contributions of leaf N and photosynthesis on N allocation, osmotic adjustors, and lipid peroxidation, as well as their internal structure and causal relationships with each other. Model fit was tested by the index of chi square value, Fisher’s P statistic (P), goodness of fit index (GFI), comparative fit index (CFI), and the root mean square error of approximation (RMSEA) [50]. SEM was analyzed using the ‘lavaan’ package in R [51].

## 5. Conclusions

Eight varieties of pecan showed different resource-utilization strategies and growth–defense tradeoffs under high temperatures. On the one hand, YLC35, with higher leaf *N*_m_ and *N*_a_ as well as the highest *P*_max_, tended to be more resource acquisitive for growing faster and yielding more, while YLC29, with the highest *N*_a_ and lower *P*_max_, preferred to be more resource conservative to enhance tolerance; on the other hand, YLC35, with lower MDA, had the highest *P*_r_, *P*_b_, and *P*_p_ to allocate more leaf N to photosynthetic apparatus for boosting PNUE, yet YLC29 with the highest MDA had the lowest *P*_r_, *P*_b_, and *P*_p_ to invest N in non-photosynthetic apparatus for improving resistance. Meanwhile, different osmotic adjustors, PRO, SPC, and SSC, were selectively regulated, suggesting that there were compensation mechanisms of tolerance to high temperatures in the eight varieties. Furthermore, N allocation was subject to a positive effect of photosynthesis but a negative effect of leaf N. There was an indirect negative effect of photosynthesis and leaf N on the lipid peroxidation via reverse regulatory processes of N allocation, indicating that N allocation had a strong influence on plant homeostatic mechanisms. Our results contribute to understanding the relationships between N allocation and plant thermotolerance, and provide useful information for the identification of varieties and crop domestication of pecan.

## Data Availability

This study did not report any data.

## Supplementary Materials

The following supporting information can be downloaded at: https://www.mdpi.com/article/10.3390/plants11212828/s1, Table S1: Eight varieties of *Carya illinoinensis* for testing.
