# Supplementary material for: Nitrogen Allocation Tradeoffs Within-Leaf between Photosynthesis and High-Temperature Adaptation among Different Varieties of Pecan (Carya illinoinensis [Wangenh.] K. Koch)
Source: Plants (Basel). 2022 Oct 24;11(21):2828. doi: 10.3390/plants11212828 (PMC9657520; doi:10.3390/plants11212828)
Supplement: Supplementary file 1 [file plants-11-02828-s001.zip › plants-1922967-supplementary.pdf]

# Supplementary Materials

# Nitrogen Allocation Tradeoffs Within-Leaf between Photosynthesis and High-Temperature Adaptation among Different Varieties of Pecan (*Carya illinoinensis* [Wangenh.] K. Koch)

**Qiwen Xu, Huichuan He, Binghui He*, Tianyang Li, Yumin Liu, Shunyao Zhu, Gaoning Zhang**

College of Resources and Environment, Southwest University, Chongqing 400715, China
*Correspondence: hebinghui@swu.edu.cn; Tel.: +86-23-68251249

**Table S1.** Eight varieties of *Carya illinoinensis* for testing.

| Variety | Number | The institution of selection and breeding | Origin |
|---|---|---|---|
| Mahan | Su R-ETS-CI-002-2011 | Institute of Botany, Jiangsu Province and Chinese Academy of Sciences | America |
| YLC10 | Zhe R-SC-CI-009-2011 | Research Institute of Subtropical Forestry, Chinese Academy of Forestry.; et al. | Zhejiang Province, China |
| YLC12 | Zhe R-SC-CI-010-2011 | Research Institute of Subtropical Forestry, Chinese Academy of Forestry.; et al. | Zhejiang Province, China |
| YLC13 | Zhe R-SC-CI-011-2011 | Research Institute of Subtropical Forestry, Chinese Academy of Forestry.; et al. | Zhejiang Province, China |
| YLC29 | Zhe R-SC-CI-013-2011 | Research Institute of Subtropical Forestry, Chinese Academy of Forestry.; et al. | Zhejiang Province, China |
| YLC35 | Zhe R-SC-CI-014-2011 | Research Institute of Subtropical Forestry, Chinese Academy of Forestry.; et al. | Zhejiang Province, China |
| YLJ042 | Zhe S-SV-CI-006-2006 | Research Institute of Subtropical Forestry, Chinese Academy of Forestry.; et al. | Zhejiang Province, China |
| YLJ5 | Zhe S-SV-CI-002-2006 | Research Institute of Subtropical Forestry, Chinese Academy of Forestry.; et al. | Zhejiang Province, China |
